# Regression of brain metastases from breast cancer with eribulin: a case report

**DOI:** 10.1186/1756-0500-6-541

**Published:** 2013-12-18

**Authors:** Hiromichi Matsuoka, Junji Tsurutani, Junko Tanizaki, Tsutomu Iwasa, Yoshifumi Komoike, Atsuko Koyama, Kazuhiko Nakagawa

**Affiliations:** 1 Department of Medical Oncology, Kinki University Faculty of Medicine, 377-2 Ohno-higashi, Osaka-Sayama, Osaka, Japan; 2 Department of Breast and Endocrine Surgery, Kinki University Faculty of Medicine, 377-2 Ohno-higashi, Osaka-Sayama, Osaka, Japan

**Keywords:** Breast cancer, Blood brain barrier, Eribulin, Brain metastases, P-glycoprotein

## Abstract

**Background:**

Eribulin is a recently approved new therapeutic option for patients with metastatic breast cancer. According to several reports, eribulin has limited ability to cross the blood brain barrier. Recently, capecitabine and eribulin have been recognized as drugs with similar application for patients with advanced breast cancer. Although there have been several case reports describing the efficacy of capecitabine against brain metastases, no report of eribulin demonstrating efficacy for brain metastases exists today.

**Case presentation:**

We describe a case of a 57-year-old Japanese woman who was diagnosed with breast cancer stage IV metastasized to multiple organs including liver and lung. After she received 3 regimens, she showed evidence of brain metastases, and whole brain radiation therapy was performed. Lapatinib and capecitabine was then administered as fourth-line chemotherapy, but the patient was hospitalized due to the exacerbation of interstitial pneumonitis and progression of brain and liver metastases. To control the systemic disease, eribulin was commenced as fifth-line chemotherapy. One month later, a significant response of brain metastases had been achieved, and this response has persisted for the last 4 months. We now describe a remarkable antitumor effect of eribulin against brain metastases from breast cancer. This case is the first report which indicates potential treatment of brain metastases using this medication.

**Conclusion:**

This report suggests that eribulin treatment may be beneficial for breast cancer patients with brain metastases progressing after whole brain radiation therapy. However, further clinical studies are warranted to determine the clinical effect of eribulin in brain metastases.

## Background

Eribulin is a recently approved new therapeutic option for patients with metastatic breast cancer [1]. In a phase III study, patients with brain metastases were excluded unless these metastases had been previously treated and stabilized [2]. Two phase II studies have excluded patients with active symptomatic brain metastases or progression of known brain metastases. However, the number of patients with brain metastases in these trials was not reported [3,4]. It is therefore important to evaluate the efficacy and safety of eribulin in patients with brain metastases. In this case, our patient experienced regression of brain metastases from breast cancer with eribulin.

## Case presentation

**Figure 1:**
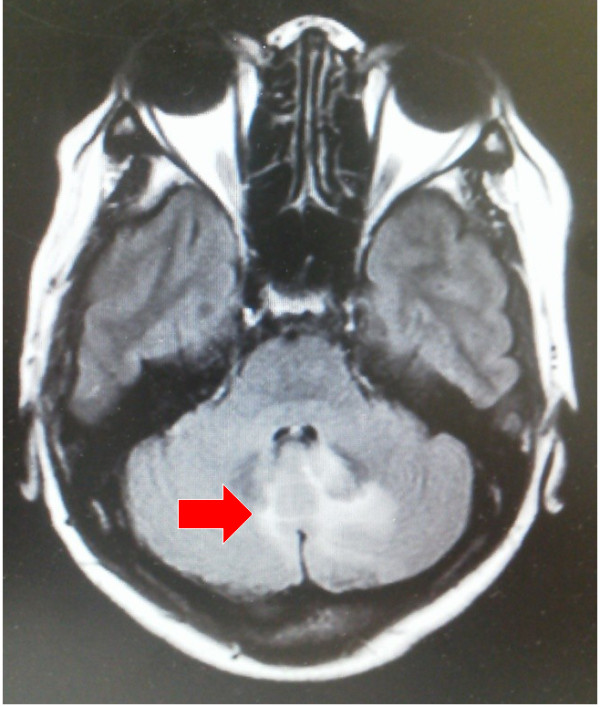
**The first evidence of brain metastases.** After 6 cycles of the 3rd-line chemotherapy, the first evidence of brain metastases was shown. The arrow indicates appearance of the new metastatic lesion.

**Figure 2:**
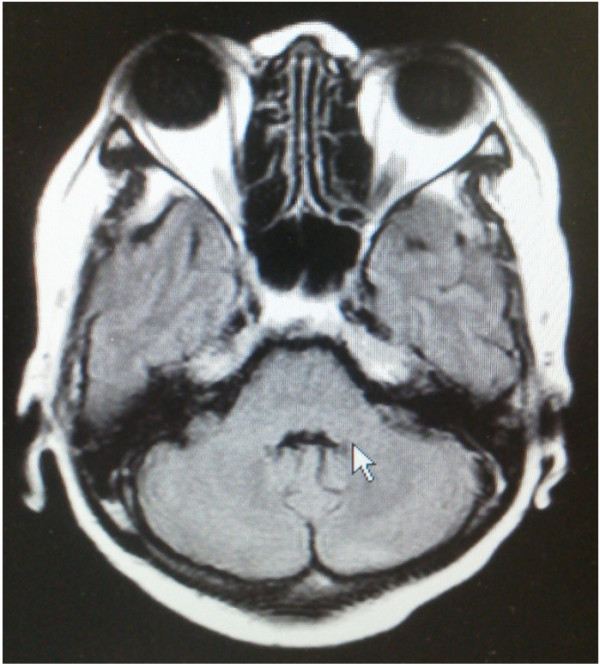
**The effect of whole brain radiation therapy.** After whole brain radiation therapy, tumor regression was observed.

**Figure 3:**
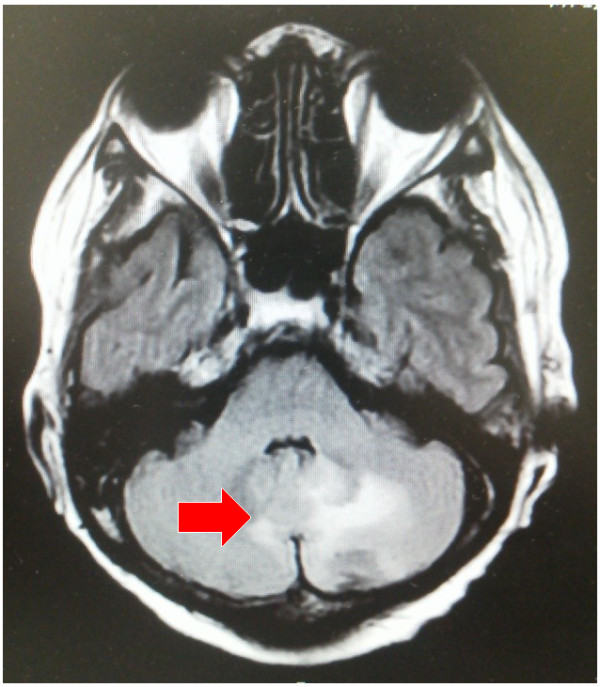
**Regrowth of the brain metastases.** 4th-line chemotherapy was administered, but patient was hospitalized again. Regrowth of the brain metastases was detected. The arrow indicates the recurrent tumor.

**Figure 4:**
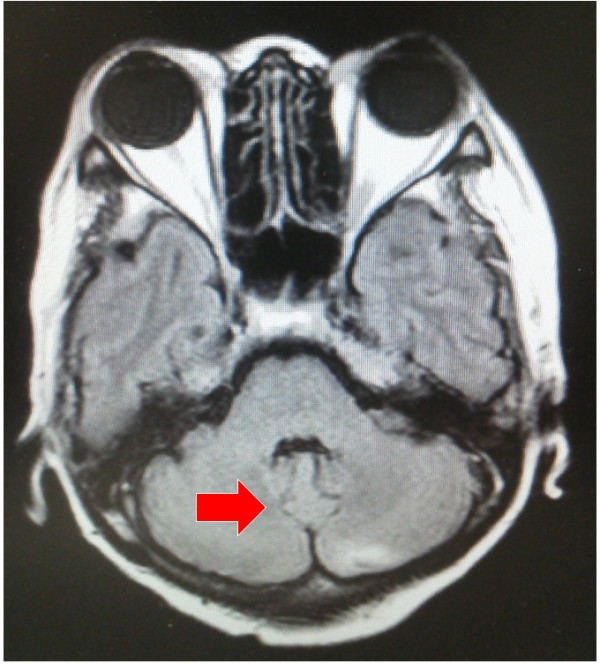
**The effect of eribulin.** After the administration of eribulin, a significant response of brain metastases had been achieved. The arrow indicates disappearance of the recurrent tumor.

A 57-year-old Japanese female former smoker presented at our hospital with a several months history of an occasional bleeding from a lump on her left breast. She was diagnosed with breast cancer with stage IV disease (cT4,N3,M1) of invasive ductal carcinoma metastasized to multiple organs including liver and lung. Pathological evaluation revealed that the tumor was negative for hormone receptor (HR) and positive for human epidermal growth factor receptor 2 (HER2) defined by immunehistochemistry and fluorescent in situ hybridization (FISH) analyses, respectively (HR-/HER2+). She received 3 cycles of first-line chemotherapy with paclitaxel (80 mg/m^2^, weekly, intravenously, 3 weeks on/1 week off) and trastuzumab (4 mg/kg loading dose and 2 mg/kg, weekly ) from October 2010 to January 2011, and the best response was a partial response. After 3 months of first-line treatment, she was diagnosed with drug-induced interstitial pneumonitis, and the administration of paclitaxel was discontinued. She was treated with oxygen and steroid-pulse-therapy, and her symptoms gradually improved as abnormal shadows on chest X-ray films were alleviated. As a second-line treatment, doxorubicin (A) and cyclophosphamide (C) (A: 40 mg/m^2^, C: 500 mg/m^2^, triweekly) was commenced in February 2011. The best response was a stable disease, and after 4 cycles of the second-line chemotherapy, the disease eventually progressed. Trastuzumab (8 mg/kg loading dose and 6 mg/kg, triweekly) and capecitabine (2,000 mg/m^2^, on days 1–14, q3w) was administered in June 2011 as a third-line treatment. Although the treatment achieved a partial response, grade 3 of hand foot syndrome (Common Terminology Criteria for Adverse Events ver 4.0) emerged after 4 cycles of the chemotherapy. We discontinued the treatment at once, and observed an improvement of the hand foot syndrome. After 6 cycles of the third-line chemotherapy, she showed evidence of brain metastases (Figure 1), and whole brain radiation therapy (WBRT) was performed in December 2011 (Figure 2). Lapatinib (1250 mg, orally, once daily) and capecitabine (2,000 mg/m^2^, on days 1–14, triweekly) was then administered as fourth-line chemotherapy in January 2012, but the patient was hospitalized 3 months later due to the exacerbation of interstitial pneumonitis and progression of brain and liver metastases (Figure 3). She was given oxygen and steroid-pulse-therapy, and the pneumonia was improved again. To control the systemic disease, eribulin (1.1 mg/m^2^, on days 1, 8, triweekly) was commenced at the patient’s request in August 2012 as fifth-line chemotherapy. One month later, a significant response of brain metastases had been achieved (Figure 4), and this response has persisted for the last 4 months.

According to preclinical study, eribulin has limited ability to cross the blood brain barrier (BBB) [5-7]. Few macromolecules are transferred into the brain because vesicular transcytosis in the endothelial cells is considerably limited and the tight junction is located between the endothelial cells. In addition, there are several types of influx or efflux transporters at the BBB, such as P-glycoprotein (P-gp), multidrug resistance-associated protein, and breast cancer resistance protein [8,9]. Permeability in the BBB is increased by WBRT. One study showed that radiation reduces P-gp expression in the brain [10]. Therefore, it is likely that radiation allows eribulin to enter into brain tissue and exert a significant anti-tumor effect. However, the effect of a standard dose WBRT persists only for 4 weeks after radiation therapy (RT) [11], and attribution of the tumor regression to radiotherapy in the present case could be very limited since 8 months have passed after radiotherapy. On the other hand, brain metastases larger than 2 mm require angiogenesis for growth and therefore newly formed capillaries in brain metastases are fenestrated and they do not have a normal functioning blood brain barrier. Eribulin is known to be substrate for P-gp mediating drug efflux in BBB. But there is a decreased expression of P-gp in the neovasculature of brain metastases when compared to normal brain tissue [12-14]. This might explain the positive effects of eribulin on brain metastases.

There has been another reported case in which the treatment of brain metastases from breast cancer with eribulin in combination with WBRT was performed [15]. The patient continued chemotherapy and received concomitant WBRT. After 3 cycles of eribulin mesylate, however, treatment was discontinued because of newly diagnosed liver metastases and progression in the lungs. Therefore, our case is the first report which shows a favorable effect on brain metastases. The treatment of eribulin was well tolerated without any high grade non-hematological toxicities, and the hematological toxicity was also acceptable.

Capecitabine and eribulin have been recognized as drugs with similar application for the patients with advanced breast cancer. Although there have been several case reports describing the efficacy of capecitabine against brain metastases from breast cancer, no report of eribulin demonstrating efficacy for brain metastases exists to date. Thus, the current case report is of value, and may help physicians determine which option to choose in treating patients with brain metastases.

## Conclusion

This report suggests that eribulin treatment may be beneficial for breast cancer patients with brain metastases progressing after WBRT. However, further clinical studies are warranted to determine the clinical effect of eribulin in brain metastases.

## Consent

Written informed consent was obtained from the patient for publication of this Case Report and accompanying images. A copy of the written consent is available for review by the Editor-in-Chief of this journal.

## Abbreviations

HR: Hormone receptor; HER2: Human epidermal growth factor receptor 2; FISH: Fluorescent in situ hybridization; WBRT: Whole brain radiation therapy; BBB: Blood–brain barrier; P-gp: P-glycoprotein; RT: Radiation therapy.

## Competing interests

The authors declare that they have no competing interests.

## Authors’ contributions

HM looked the patient, conceived of the study and drafted the manuscript. JT looked the patient and revised the manuscript critically. JT looked the patient and conceived of the study. TI looked the patient and revised the manuscript critically. YK revised the manuscript critically and participated in its design and coordination. AK conceived of the study, and participated in its design and coordination. KN looked the patient and participated in its design and coordination. All authors read and approved the final manuscript.

## Authors’ information

Hiromichi Matsuoka, MD: Oncologist, Psycho-Oncologist, Associate Professor of Department of Medical Oncology, Kinki University Faculty of Medicine, Osaka, Japan.

Interests: Chemotherapy of breast cancer, Palliative care, Psycho-Oncology.

Junji Tsurutani, MD: Oncologist, Associate Professor of Department of Medical Oncology, Kinki University Faculty of Medicine, Osaka, Japan.

Junko Tanizaki, MD: Oncologist, Associate Professor of Department of Medical Oncology, Kinki University Faculty of Medicine, Osaka, Japan.

Tsutomu Iwasa, MD: Oncologist, Associate Professor of Department of Medical Oncology, Kinki University Faculty of Medicine, Osaka, Japan.

Yoshifumi Komoike, MD: Surgeon, Proffesor of Department of Breast and Endocrine Surgery, Kinki University Faculty of Medicine, Osaka, Japan

Atsuko Koyama, MD: Psycho-Oncologist, Professor of Department of Medical Oncology, Division of Psychosomatic Medicine, Kinki University Faculty of Medicine, Osaka, Japan.

Kazuhiko Nakagawa, MD: Oncologist, Professor of Department of Medical Oncology, Kinki University Faculty of Medicine, Osaka, Japan.
